# 

**Published:** 1900-11-24

**Authors:** 


### NOTICE TO CORRESPONDENTS.

All MSS., Letters, Books for Review, and other matters intended for the Editor should be addressed THE EDITOR, "THE HOSPITAL," THE HOSPITAL BUILDING, 28 & 29 SOUTHAMPTON STREET, STRAND, W.C.

The Editor cannot undertake to return rejected MS., even when accompanied by stamped directed envelope.

**Notice to Advertisers and Subscribers.**

All Advertisements, Orders for Copies of THE HOSPITAL, and Business Communications should be addressed to **THE MANAGER (Not to the Editor)**, THE HOSPITAL, 28 & 29 Southampton Street, Strand, London, W.C.

The Cost of Subscription, post free, is as follows:—Per week, 2½d.; per quarter, 2s. 8d.; per half-year, 5s. 3d.; per annum, 10s. 6d. Foreign:—Per annum, 15s. 2d., payable, in all cases, in advance.

**NOTICE.—Vol. XXVIII. of "The Hospital" (April to September, 1900), in handsome cloth binding, is now on sale at the Office of this Journal, price, 7s. 6d. Cases for binding the Numbers contained in this Volume 1s. 6d. Index to Vol. XXVIII., 2½d., post free.**

**The Monthly Part for December will be ready on December 1. Price 6d., by post 8d.**

### BOOKS RECEIVED.

Sampson Low, Marston and Co.

"Twentieth Century Practice of Modern Medical Science." Edited by Thomas L. Stedman, M.D. Vol. XX.

Longmans, Green and Co.

"The Essentials of Practical Bacteriology." By H. J. Curtis, B.S. and M.D. Lond., F.R.C.S.

Warne and Co.

"The Mystery of Lady Place." By Percy J. Brebner.

National Union Publishing Co.

"Children of Scorn." By Lady Cook (*née* Tennessee Claflin).

Jarrold and Sons.

"The Norfolk and Norwich Hospital, 1770 to 1900." By Sir Peter Eade, M.D. Lond.

Baillière, Tindall and Cox.

"The Microscopy of the More Commonly Occurring Starches." By Hugh Galt, M.B., C.M., D.P.H.

Gann and Co., Boston, U.S.A.

"Laboratory Direction for Beginners in Bacteriology." By Neranus A. Moore, B.S., M.D.

### Scraps and Gleanings.

The post of Chemical Lecturer at St. Thomas's Hospital has become vacant by the resignation of Prof. W. R. Dunstan.

Since the opening twelve months ago of the Dublin City Hospital for Diseases of the Skin 2,000 cases have been treated.

The new additions to the out-patient department at the Sheffield Royal Infirmary have cost £3,000. The building of the new operation theatre has cost £7,000. The architect is Mr. Keith D. Young.

The Hospital Saturday Collection in aid of the Bridgnorth Infirmary has been a record one this year, amounting to £119. Here at any rate War Funds have not sapped the source of hospital revenues.

The foundation stone of the Isle of Thanet Isolation Hospital was recently laid by the Right Hon. J. Lowther. The site of the new buildings is between Margate and Ramsgate. The architects are Messrs. Langham and Osborne.

The site for the new Sutton Cottage Hospital has been finally decided upon by the Committee. The funds for the purchase of the site have been provided by Mr. Ralph C. Forster, J.P., and Mr. Passmore Edwards has promised to pay for the erection.

At the recent quarterly meeting of the Governors of the Oldham Infirmary the following resolution was carried:—"That an extension of the Infirmary on the lines of the plans prepared by the extension committee of the Governors of the Royal Infirmary and approved by the Jubilee Committee in 1899 is urgently needed." £10,000 is now available for this purpose, the estimate for the suggested alterations being £15,000.

The report of the committee of the Birmingham Hospital Saturday Consultative Institution shows that during the period the institution has been opened, 21 weeks, 222 patients have attended for consultation, the amount received in consultation fees being £116 11s., the expenditure in connection with fitting up and furnishing the consulting rooms was £213 17s. 3d., and the necessary establishment expenses amount to £161 odd. The total deficiency of income is stated to be £343 odd, but the committee believe that before long this debit balance will disappear and the institution become self-supporting.

